# The London Hospital and the National Pension Fund for Nurses

**Published:** 1889-08-17

**Authors:** G. Q. Roberts

**Affiliations:** secretary. London Hospital


### The London Hospital and the National Pension Fund for Nurses.

It has been decided by the House Committee :

1. That the hospital is prepared to assist all sisters, staff nurses, or private nurses of the London Hospital under forty years of age, who may desire to join the "National Pension Fund for Nurses," by paying one half of their annual premium whilst they remain in the service of the hospital.

2. That these premiums shall be paid into the Returnable Fund of the "National Pension Fund for Nurses" on Table B, to secure a pension of £22 10s. (minimum) to commence at the age of fifty years.

3. That sisters and nurses withdrawing their own half of the premium whilst in the service of the hospital, or within twelve months after leaving, will forfeit the amount paid to their name by the hospital, but that after that date the amount will be considered as belonging to them.

4. That any sisters and nurses who are discharged by the hospital, or who leave within twelve months of their appointment on the permanent staff, will forfeit the amount paid by the hospital to their name in the National Pension Fund.

5. That in every instance money which has been paid by the London Hospital and forfeited in the Pension Fund shall remain in the fund for the benefit of London Hospital sisters and nurses.

6. That probationers in the London Hospital Training School for Nurses who may join the "National Pension Fund for Nurses" under Table B, to secure a pension of £22 10s. (minimum) at fifty years of age shall, if appointed on the permanent staff on completion of their training, receive from the hospital one-half of the premiums already paid by them to the National Pension Fund.

7. That the committe are prepared to consider what help should be given to sisters and nurses over forty years of age, with a view to making such arrangements as may seem best in each individual case.

8. That those desirous of joining the pension fund on these conditions shall send in their names to the matron.

Printed by order of the House Committee,

G. Q. Roberts, secretary.

London Hospital, July, 1889.

